# Meetings of Societies

**Published:** 1912-06

**Authors:** 


### MEETINGS OF SOCIETIES.

### Bristol Medico-Chirurgical Society.

*March 13th*, 1912.

Mr. C. A. Morton, President, in the Chair.

A discussion on "**The Intracranial Complications of Ear Disease**" (see p. 97).

---

*May 8th*, 1912.

Mr. C. A. Morton, President, in the Chair.

Dr. E. C. Williams: (1) A case of **Extensive hairy mole,**

(2) a case of **Infantilism,** and (3) a case of **Precocious development.** The last-mentioned patient was a boy not yet six years old. He was 4 ft. 2 in. in height, weighed 5 stone 2 lb., the weight of a normal child of eleven. He had the beginning of a moustache, pubic hair, and spoke with an adult type of voice. Such cases had occurred in connection with supra-renal and testicular tumours, and in girls with solid ovarian tumours. The patient had no signs of a tumour.

Dr. Edgeworth read a note on the **Innervation of the palate.** The levator palati is supplied by the vagus-accessorius nerve through the pharyngeal plexus. The muscle is a derivative of the pharyngeal musculature embryologically, and is correspondingly innervated. With regard to the tensor palati, anatomists have said that filaments from the mandibular portion of the Vth nerve can be traced to this muscle; but removal of the Gasserian ganglion has not given uniform results in the hands of different observers. The speaker had traced the development of the muscle, and found it came from the internal face of the internal pterygoid muscle. It is therefore one of the masticatory group of muscles. Some months ago Sir Victor Horsley divided the Vth nerve in two monkeys, and found that the tensor palati muscle subsequently wasted. This shows that the innervation was from the Vth. The diverse clinical results from the removal of the Gasserian ganglion were explicable on the assumption that the tensor muscle had not much influence on the normal shape of the palate.

Mr. Hey Groves read a paper on "**Some clinical and experimental observations on the treatment of fractures of long bones, with special relation to the use of intra-medullary pegs.**" Special pegs used by the speaker, with a gauge, drills and wires for use in applying the pegs, were exhibited, and the method of inserting the pegs illustrated by lantern slides. In certain cases he considered the use of these pegs was very advantageous for securing correct alignment and stability until the fracture was repaired. With the pegs in position no external splints were required.—The President said he had hitherto hesitated to use intra-medullary supports in factures because he thought they would be so difficult to remove later if trouble arose from their presence.—Mr. Groves replied that he had removed the pegs in three cases—all cases of comminuted fracture, really unsuitable for the application of the pegs—and he had not found it a difficult proceeding. With aseptic results the pegs gave no trouble.

Mr. H. Chitty read a paper on "**The artificial production of pneumothorax in phthisis**" (see p. 141). The paper was discussed by Drs. Kennedy and Nixon and Mr. Mole.

The Hon. Secretary read the following communication from a Medical Correspondent :—

"My dear Dr. Nixon,

"I am only too pleased to tell you what I can about my pneumothorax experience, for though my 'good' lung is so damaged that I cannot immediately reap the full benefit of the treatment, I can certainly testify to substantial im provement, with a reasonable hope for still better things in the future. Without boring you with too many irrelevant details, I may perhaps remind you that I began to cough in January, 1909; had first hæmoptysis in April, 1909; and since April, 1910, I have been nearly always in bed with a temperature between about 99° in the mornings, and 100° to 101° at night. Profuse thick expectoration, about 10 oz. in 24 hours, and containing, besides numerous T.B.'s, a large and varied assortment of cocci. Physical signs have always been very slight, and in response to the method of feeding in vogue in the sanatorium, my weight increased from 12 stone to 14 stone 7 lb. X-rays long ago showed that the right lung was much more extensively involved than the left. On January 29th, 1912, Dr. Lillingston gave me my first dose of nitrogen—300 c.c. I felt no inconvenience whatever. The next day I had 600 c.c.; and after that I certainly felt considerable oppression of breathing for about forty-eight hours.

"Since then, at intervals of eight or nine days, I have had doses of 700 c.c., 750 c.c., then 300, then 1,000, then 300, then 1,000 again, and on the last three occasions 500 only. After the large doses I was uncomfortable, restless, and feeling 'full of wind' (not much wonder!), but after a few days that passed off. The first effect on the expectoration is to increase it, owing no doubt to its being squeezed out of the compressed lung; and, owing to a want of *vis a tergo*, the difficulty of expelling it is also increased, but after the first twenty-four hours it becomes less. The temperature is raised for the first day or two—I think probably due to increased absorption owing to pressure, an auto-inoculation in fact—but its general level has dropped to normal. I have been able to take walks of nearly two miles; but my left lung, the only one I have left (if I may be allowed to say so) is not a very good one, so I have to be careful and move slowly.

"On the whole, I think the operation deserves a wider recognition than it has hitherto received. It should not be reserved only for cases which are otherwise hopeless; but on the other hand I do not think it suitable for those who are able to get about, and for whom the possibility of cure by graduated exercise (or graduated labour) under sanatorium conditions, is not nearly exhausted. One must, I think, hesitate before condemning any patient to rely entirely on one lung, though of course at any time the compressed lung may be allowed to expand again.

"I must, however, qualify my remark about the unsuitability of the operation for a patient 'able to get about.' I had in my mind a man able to walk his six or eight miles a day with one-sided disease which one might be tempted to put to rest; but for a patient only able to move about very gently and liable to 'auto-inoculations' on very slight exertion, and especially if there has been hæmoptysis, I think the operation is clearly indicated. What I think it is most important to recognise is that the treatment is *not* to be regarded as a sort of forlorn hope, but one which gives every prospect of success, and is singularly free from risk when properly carried out. There are four cases here just now, and likely to be a fifth, I believe, if they can get a needle into the pleural cavity. All doing well.

"As to the pressure, it may be of interest to note that in my case, at the commencement of each 'refill,' the manometer shows a minus pressure at end of ordinary inspiration of 10 centimetres of water (*i.e.* 5 above and 5 below zero), and a positive pressure at end of expiration of about 1 centimetre. At the conclusion of the performance, after 500 c.c. of nitrogen have been allowed to flow in, the inspiratory minus pressure is equal to about 3 c.m. and the expiratory positive pressure = 4. That is as much as I can stand with any comfort. No doubt in cases where the available pleural space is much circumscribed by adhesions a greater positive pressure can be borne; but in my case the pressure is being exerted on the whole surface of the lung, and probably already produces a fair amount of displacement of everything over towards the left.

"The treatment is long and somewhat irksome, necessitating a small operation, which, by the way, is a mere trifle, hardly to be ranked as an 'operation' at all, but which has to be repeated by some skilled person every ten days until the lung is healed—perhaps for twelve months or more. These considerations seem to me to limit its indication to cases of troublesome hæmoptysis and cases with chronic septic absorption, where one lung is at least comparatively sound. In these cases, and they are not a few, it is, I think, likely to prove invaluable, and lucky is he who in such a plight has not an adherent pleura."

E. MARIETTE, M.B. Lond.

J. LACY FIRTH.
J. A. NIXON, *Hon. Sec.*

